# Breast cancer treatment and sexual dysfunction: Moroccan women's perception

**DOI:** 10.1186/1472-6874-11-29

**Published:** 2011-06-13

**Authors:** Yassir Sbitti, Habiba Kadiri, Ismail Essaidi, Zouhour Fadoukhair, Soussane Kharmoun, Khaoula Slimani, Nabil Ismaili, Mohammed Ichou, Hassan Errihani

**Affiliations:** 1 Medical oncology Departement, University Military Hospital of Instruction, Rabat, Morocco; 2 Department of Cytopathology, diagnostic center, Rabat, Morocco; 3 Medical oncology Departement, National Institute of Instruction, Rabat, Morocco

## Abstract

**Background:**

This exploratory prospective study evaluated women's responses to questions that asked them to describe how their body image and sexual functioning had changed since their breast cancer diagnosis to treatment.

**Methods:**

A questionnaire concerning body image scale and various sexual problems experienced after diagnosis and treatment was anonymously completed by 120 women in the outpatient clinic of our hospital's Division of medical Oncology. To be eligible, subjects had to be sexually active and had histology proven breast cancer. They also had to have received treatment for breast cancer.

**Results:**

100% of participants have never spoken with their doctor about this subject. 84% of the participants continued sexual activity after treatment, but there was an increase in the incidence of sexual functioning problems which resulted in a slight reduction in the quality of their sex lives. 65% of the women experienced dyspareunia followed by lubrication difficulties (54%) and the absence or reduction of sexual desire (48% and 64%, respectively) while, 37% had lack of satisfaction (37%). Female orgasmic disorder and brief intercourse and arousal were reported respectively by 40% and 38% of the subjects. The sexual dysfunctions were absent before diagnosis and management of breast cancer in 91.5% subjects and of these 100% subjects complained of a deterioration of the symptomatology after the various treatments. 90% of the dysfunctions were observed after chemotherapy, 9% after surgery and 3% after radiotherapy; none of the subjects indicated the onset of dysfunctions to have been associated with hormonotherapy. 100% expressed not having received sufficient information about how the disease and treatment (including surgery) might affect their sexual life.

**Conclusion:**

Breast cancer and its treatment may result in significant difficulties with sexual functioning and sexual life. Addressing these problems is essential to improve the quality of life of Moroccan women with breast cancer.

## Background

The breast is often referred to as the body part that is most strongly associated with women's femininity, maternal role, and sexuality [1]. When referring to sexuality, a distinction should be made between the terms sexuality and sexual functioning. "Sexuality is a personal expression of one's self and one's relationship with others" [2]. "Sexuality encompasses feelings about one's own body, the need for touch, interest in sexual activities, communication of one's needs to a partner, and the ability to engage in satisfying sexual activities" [3]. In contrast, sexual functioning refers to areas of functioning such as vaginal lubrication, frequency of sexual activity and breast sensitivity [4]. Based on the lack of research in sexual functioning issues among Moroccan female cancer survivors and on views in the research that interpret ''sexual activity'' as limited to penis-in-vagina intercourse, the authors decided to focus on the sexual impact of breast cancer treatments on Moroccan women. Changes in the breast may not necessarily interfere with women's physical ability to have sexual intercourse; however it is strongly associated with sexual well-being, body image and feminine identification [5,6].

The aim of this study was to prospectively evaluate body image scale, and the impact of breast cancer therapy on women's sexuality from Morocco.

## Methods

### Participants

From December 2009 to February 2010, after obtaining informed consent, 120 women diagnosed with breast cancer were recruited from a medical oncology department at University Military Hospital of Instruction at Rabat (Morocco). In order to ensure patients privacy all interviews were conducted by the female medical physician in a quiet and comfortable environment. They were selected on the basis of inclusion criteria: histologic proven breast cancer, being married and sexually active, having radical or conserving breast Surgery and received medical treatment. Exclusion criteria were: having a history of pelvic surgery.

### Instruments

The Body Image Scale (BIS), designed for the assessment of body image in cancer patients, concerning impact of treatment on self-consciousness, physical and sexual attractiveness, femininity, satisfaction with body and scars, body integrity, and avoidance behaviour scored according to the original article [7].

The Female Sexual Function Index (FSFI) consists of 19 items constituting six subscales (desire, arousal, lubrification, orgasm, satisfaction, pain) scored according to the original article [8]. Items about sexual activity are included.

### Data Collection and Procedure

Both questionnaires were translated from English to Arabic language. We used a method involving translation and back-translation procedures, in collaboration with professionals, psycho-oncologists and a translator/linguist. A pilot test was performed. A random sample of 15 patients was studied. All agreed to participate and were asked to complete the questionnaire. All patients and their accompanying female's persons found the questionnaire straightforward and easy to complete. Data were collected using self-administered questionnaires including some demographic data, body image scale and Female sexual index Function. Illiterate and low levels of education patients were helped by nurse oncology and female member of her family. The women were in a separate and quiet room of medical oncology Department when they marked the questionnaires. All of the participants completed the questionnaires. 15 patients were already receiving some form of active treatment as part of their overall cancer management.15 patient's undergone surgical treatment. 15 patient's chemotherapy, 15 are receiving radiotherapy and 8 of the total number of patients have received hormone therapy. The score of Body image scale and FEMALE SEXUAL INDEX FUNCTION were included in study results

## Results

### Demographic characteristics

Demographic data of the women in the study were shown in Table 1.

**Table 1:** Participants Demographics characteristics (N = 120)

Age at diagnosis (years)		
Mean (+/-) S.D.	45.3 (range 25-58 years), 5.24	
Educational level N (%)		
Illiterate	40(34%)	
Elementary	20(16%)	
Junior college	45(37.5%)	
High school	15(12.5%)	

Martial status N (%)	before	After
Married	120 (100%)	100 (84%)
Divorced	0%	20(16%)

Stage of breast cancer N (%)		
Localized	110(91.6%)	
Metastasis	10 (8.4%)	

Financial dependence N (%)		
Own work	20(16%)	
Partner	100(84%)	

Breast cancer Treatment N (%)		
Type of surgery:		
Radical Mastectomy/	80(66.5%)	
Breast Conserving Surgery	40(33.5%)	
Chemotherapy	120(100%)	
Radiotherapy	110 (91.5%)	
Hormone therapy	75(62.5%)	

The patient's' mean age at diagnosis was 45.3 S.D 5.24 (range 25-58years). The levels of education attained were elementary in 16%, junior college in 5%, high school in 12.5%, 34% of participants was illiterate. 100% of the women were married before breast cancer management and 20% divorced after breast cancer. Most of patients had undergone breast surgery (radical mastectomy 66.5%, lumpectomy 33.5%) for relatively early stage breast cancer. All of them had some kind of adjuvant therapies such as intra-venous chemotherapy 100%, radiation 91.5%, and hormone therapy 75%. 10 patients had metastasis disease received chemotherapy with target-therapy (trastuzumab or bevacizumab). All of participants had a sexual partner before breast cancer diagnosis.

**Body image scale**: (Table 2)

**Table 2:** Body Image Scale (N = 120)

	Not at all0		A little 1		Quite a Bit 2		Very Much3	
Scale item	N	%	N	%	N	%	N	%
Self conscious	0	0	20	16.5	15	21	75	62.5
Less physically attractive	25	21	15	12.5	15	12.5	65	54
Dissatisfied with apparence	0	0	23	19	18	15	79	66
Less feminine	98	81.5	7	5.8	12	10	3	2.5
Difficult to see sell nacked	10	8.5	22	18.5	8	6.5	80	66.5
Less sexually active	10	8.5	35	29	20	16.5	55	46
Avoid people	10	9	32	26.5	10	9	68	55.5
Body less whole	10	8.5	35	29	20	16.5	55	46
Dissatisfied with body	10	8.5	22	18.5	8	6.5	80	66.5
Dissatisfied with scar	20	16.5	23	19.5	7	5.8	70	58.2

50% participants women experienced to or more body image problems some of the time (33%), or at least one problem much of the time (17%). Among sexually active women, greater body image problems were associated with mastectomy, hair loss, fatigue, asthenia and changes in hormonal status (chemically induced menopause) from chemotherapy, concern with weight gain or loss, poorer mental health, lower self-esteem. There was no significant difference in body image scores between patients undergoing mastectomy or BCT apart from a general feeling of physical unattractiveness in mastectomy patients (4.6 versus. 2.4; P 0.03).

**Female Sexual Index Function**: (Table 3)

**Table 3:** Female Sexual Index Function (FSIF) (N = 120)

	Questions	Factor	Score Mean +/- SD
Desire	1.2	0.6	3.6 (1.9)

Arousal	3.4.5.6	0.3	3 (2.5)

Lubrification	7.8.9.10	0.3	3.9 (1.6)

Orgasm	11.12.13	0.4	1.6 (2)

Satisfaction	14.15.16	0.4	3.6 (0.9)

Pain	17.18.19	0.4	5 (1.9)

Full Scale score			17.7 (12)

The most frequent sexual dysfunctions were dyspareunia (65%) followed by lubrication difficulties (54%) and the absence or reduction of sexual desire (48% and 64%, respectively). About one third of subjects complained of inhibited female orgasm (40%), lack of satisfaction (37%), brevity of intercourse and arousal (38%). The sexual dysfunctions were absent before diagnosis and management of breast cancer in 91.5% and of these 100% complained of a deterioration of the symptomatology after the various treatments. 78% of the subjects indicated that they had never before suffered from sexual dysfunctions. 19% of the dysfunctions were observed after chemotherapy, 9% after surgery and 3% after radiotherapy; none of the subjects indicated the onset of dysfunctions to have been associated with hormonotherapy. All participants indicated that they had never before discussed from sexual dysfunctions with medical or paramedical team manager. 100% of subjects expressed not having received sufficient information about how the disease and treatment (including surgery) might affect their sexual life. In this sample we found no statistically significant correlation between the presence of sexual dysfunctions and other variables (age, level education, financial dependence, stage disease, type of surgery, type of medical therapy). This absence of correlation is probably due to the sample size and its general homogeneity, and therefore the data cannot be used to make generalizations about larger samples.

## Discussion and Conclusion

Over the past thirteen years a great deal of effort has been expended to enhance the possibility of early diagnosis and improve the treatment of breast cancer, and problems related to the disease have been extensively investigated. It is now clear that the psychological problems most frequently observed in these patients are depression, body image-associated hypochondria and sexual dysfunction [9,10]. Our study, aimed to assess in treated breast cancer Moroccan women the subjective disturbance of sexual dysfunction that often is not expressed and that contributes to further lowering the quality of life. The interviews revealed a number of notable findings regarding sexuality after breast cancer in the Moroccan context. The sexual impact of having cancer and its treatments have long been a taboo topic in clinical settings in Morocco. The sexual aspects of having cancer have long been neglected in medical practice. There are a number of barriers to the discussion of sexual issues by patients and professionals. Difficulties related to sexuality and sexual functioning was common and occurred since their breast cancer diagnosis to treatment. The majority of informants revealed that they had experienced sexual dysfunction and physical discomfort caused by the breast cancer treatment. Patients reported worse sexual functioning, characterized by greater lack of sexual interest, inability to relax and enjoy sex, difficulty becoming aroused, and difficulty reaching the orgasm. Regarding personal and sexual relationships, 98% had an important personal relationship (partner) and 84% were effectively sexually active; following diagnosis and various treatments of breast cancer. 60% of the subjects had ceased sexual relations and 20% divorced. However, we were not able to compare the data from this study regarding sexual problems with those of other groups (normal population or samples of subjects with chronic disease) since to date no epidemiological studies of sexual behaviour and distribution of sexual dysfunctions in the normal population have been performed in Morocco. Nor was it possible to make a comparison between the data obtained from this study and that which had emerged from research carried out in other countries because of cultural, social, economic and moral variables which enormously condition sexual behaviour. Nevertheless, patients of this study had lower scores in the all body image subscales. This finding is comparable with other studies [11-14]. Howighorst-Knapstein and al also found that, mastectomy resulted in lower sexual desire and changes in body image [15]. Bakwell & Volker also showed that all types of treatment for breast cancer had a significant impact on body image and menopausal status and finally results in sexual problems [16]. In Summary, breast cancer affects many aspects of a Moroccan woman's sexuality, including changes in physical functioning and in perception of femaleness but this study has some limitations. The first limitation of this study is the sample structure which was limited to breast cancer women and didn't study healthy women with these problems. The data obtained from this study must be interpreted with caution since they reflect the limited methodology with which the research was conducted. The sample seems to be small and not representative enough to allow us to extrapolate the data for a larger population. The second limitation of this study is the missing role of husband/partner in the study which can have a very important role in patient life. However, this study show that the onset of sexual dysfunctions in concomitance with and after treatment of breast cancer is frequent and that such dysfunctions noticeably compromise the quality of sex life and does sufficiently address a problem that needs to be investigated in greater depth. Although it is not possible to make distinctions, it appears that the most numerous dysfunctions are those which originate easily from compromises in physical state (dyspareunia and lubrication difficulties) while the fewest dysfunctions were of a psychological nature (the absence or reduction of sexual desire, difficulty to reach orgasm and brevity of intercourse). These disturbances, even though they noticeably reduce the quality of sex life, do not compromise it completely, as the majority of subjects remain sexually active.

Healthcare professionals should include an assessment of the effects of medical and surgical treatment on the sexuality of breast cancer survivors. Oncology nurses can recognize medical outcomes and sexual issues and they are best suited to offer specific and meaningful support that is adapted to the specific demands of the patients and that considers the specific characteristics of patients and their partners. Physician should pay more attention to discussing sexual dysfunctions with the patient as part of the side effects experienced during the programme of treatment for breast cancer. In order to deal with women's sexual issues appropriately, it is important to prepare a secure environment in the hospital to discuss sexual problems with patients. It is also necessary to promote understanding about sexual issues among health care providers in general as well as increasing the number of sex therapist and counsellors.

## Competing interests

The authors declare that they have no competing interests.

## Authors' contributions

SY conceived the original idea for the study design and drafted the manuscript. KH, KA, EI, enrolled patients, participated in data acquisition, and revised the manuscript SK, ZF, MF, NI participated in data collection and revised the manuscript. MI and HE coordinated the study and gave critical comments on the draft manuscript. All authors read and approved the final manuscript.

## Pre-publication history

The pre-publication history for this paper can be accessed here:

http://www.biomedcentral.com/1472-6874/11/29/prepub
